# Supplementary material for: Effect of Ursolic and Oleanolic Acids on Lipid Membranes: Studies on MRSA and Models of Membranes
Source: Antibiotics (Basel). 2021 Nov 11;10(11):1381. doi: 10.3390/antibiotics10111381 (PMC8615140; doi:10.3390/antibiotics10111381)
Supplement: Supplementary file 1 [file antibiotics-10-01381-s001.zip › antibiotics-1435615-supplementary.pdf]

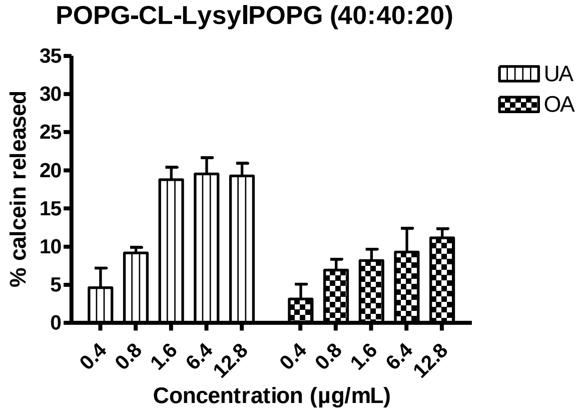

Figure S1. Effect of UA (Ursolic acid) (vertical lines) and OA (Oleanolic acid) (lattice) on calcein release in lipid vesicles (POPG:CL:Lysylphosphatidylglycerol, 40:40:20). The ordinate shows the percentage of calcein released expressed in percentage of the effect induced by 2% Triton X-100 (considered as 100 %). Data (SEM) are representative of three independent experiments in triplicate. POPG (1-Palmitoyl-2-oleoyl-*sn*-glycero-3-phosphoglycerol), CL (cardiolipin)

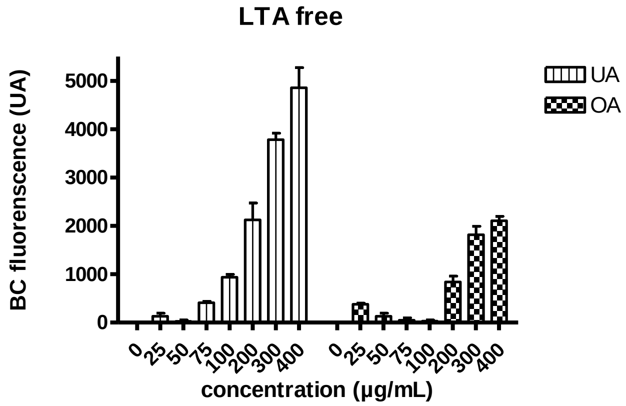

Figure S2. BODIPY™-TR-cadaverine (BC) displacement from its binding to LTA induced by increasing concentrations of selected compounds UA (Ursolic acid) (vertical lines) or OA (Oleanolic acid) (lattice). The data represent the mean ± SEM of three separate experiments.
